# Caspase-8 promotes scramblase-mediated phosphatidylserine exposure and fusion of osteoclast precursors

**DOI:** 10.1038/s41413-024-00338-4

**Published:** 2024-07-11

**Authors:** Brenda Krishnacoumar, Martin Stenzel, Hilal Garibagaoglu, Yasunori Omata, Rachel L. Sworn, Thea Hofmann, Natacha Ipseiz, Magdalena A. Czubala, Ulrike Steffen, Antonio Maccataio, Cornelia Stoll, Christina Böhm, Martin Herrmann, Stefan Uderhardt, Robert H. Jenkins, Philip R. Taylor, Anika Grüneboom, Mario M. Zaiss, Georg Schett, Gerhard Krönke, Carina Scholtysek

**Affiliations:** 1 Department of Internal Medicine 3 - Rheumatology and Immunology, Friedrich-Alexander University Erlangen-Nürnberg (FAU) and Universitätsklinikum Erlangen, 91054 Erlangen, Germany; 2 Deutsches Zentrum für Immuntherapie (DZI), Friedrich-Alexander University Erlangen-Nürnberg (FAU) and Universitätsklinikum Erlangen, 91054 Erlangen, Germany; 3 Department of Biopsectroscopy, Leibniz Institut für Analytische Wissenschaften-ISAS-e.V., Bunsen-Kirchhoff-Str. 11, Dortmund, 44227 Germany; 4 Medical Faculty, University Hospital, University Duisburg-Essen, Essen, 45147 Germany; 5 Systems Immunity Research Institute, Heath Park, Cardiff University, Cardiff, CF14 4XN UK; 6 Optical Imaging Competence Centre (FAU OICE), Exploratory Research Unit, Friedrich-Alexander University Erlangen-Nürnberg (FAU), Erlangen, Germany; 7 Division of Infection & Immunity, Heath Park, Cardiff University, Cardiff, CF14 4XN UK; 8 Deutsches Rheuma-Forschungszentrum Berlin, Berlin, Germany; 9 Department of Rheumatology and Clinical Immunology, Charité - Universitätsmedizin Berlin, Berlin, Germany

**Keywords:** Bone, Physiology

## Abstract

Efficient cellular fusion of mononuclear precursors is the prerequisite for the generation of fully functional multinucleated bone-resorbing osteoclasts. However, the exact molecular factors and mechanisms controlling osteoclast fusion remain incompletely understood. Here we identify RANKL-mediated activation of caspase-8 as early key event during osteoclast fusion. Single cell RNA sequencing-based analyses suggested that activation of parts of the apoptotic machinery accompanied the differentiation of osteoclast precursors into mature multinucleated osteoclasts. A subsequent characterization of osteoclast precursors confirmed that RANKL-mediated activation of caspase-8 promoted the non-apoptotic cleavage and activation of downstream effector caspases that translocated to the plasma membrane where they triggered activation of the phospholipid scramblase Xkr8. Xkr8-mediated exposure of phosphatidylserine, in turn, aided cellular fusion of osteoclast precursors and thereby allowed generation of functional multinucleated osteoclast syncytia and initiation of bone resorption. Pharmacological blockage or genetic deletion of caspase-8 accordingly interfered with fusion of osteoclasts and bone resorption resulting in increased bone mass in mice carrying a conditional deletion of caspase-8 in mononuclear osteoclast precursors. These data identify a novel pathway controlling osteoclast biology and bone turnover with the potential to serve as target for therapeutic intervention during diseases characterized by pathologic osteoclast-mediated bone loss.

**Figure:**
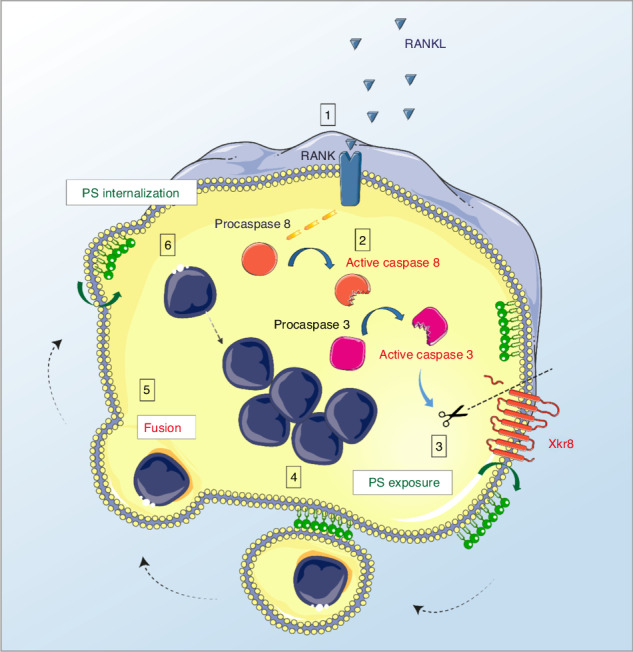
**Proposed model of osteoclast fusion regulated by caspase-8 activation and PS exposure**. RANK/RANK-L interaction. Activation of procaspase-8 into caspase-8. Caspase-8 activates caspase-3. Active capase-3 cleaves Xkr8. Local PS exposure is induced. Exposed PS is recognized by the fusion partner. FUSION. PS is re-internalized.

**Proposed model of osteoclast fusion regulated by caspase-8 activation and PS exposure**. RANK/RANK-L interaction. Activation of procaspase-8 into caspase-8. Caspase-8 activates caspase-3. Active capase-3 cleaves Xkr8. Local PS exposure is induced. Exposed PS is recognized by the fusion partner. FUSION. PS is re-internalized.

## Introduction

Bone turnover is controled by the continuous activity of bone-forming osteoblasts and bone-resorbing osteoclasts.^1^ Mature and active osteoclasts represent multinucleated syncytia that form via the fusion of mononuclear cells originating from embryonic erythro-myeloid precursors or blood monocytes.^2^ The cytokine RANKL serves as a master regulator of osteoclast differentiation and induces a series of transcriptional events in RANK-expressing precursor cells,^3,4^ which include the activation of the transcription factors c-Fos and NFATc1 that in turn control the transcription of different gene expression modules and the subsequent process of osteoclastogenesis.^5^

Although osteoclast differentiation has been studied in detail, the molecular mechanisms that control fusion of osteoclast precursors (OCPs) are still incompletely understood. Various surface molecules such as integrins and receptors including DC-STAMP and OC-STAMP were shown to modulate or contribute to cellular fusion, but their exact role during this process as well as their ligands and interaction partners remain largely elusive.^6,7^ In our current study, we identify RANKL-mediated and non-apoptotic activation of caspase-8 and downstream effector caspases as early key events promoting the fusion of OCPs. Caspase activation triggered caspase-mediated cleavage and activity of the phospholipid scramblase Xkr8, a process that disturbed the polarity of the plasma membrane and thereby essentially aided cellular fusion. Efficient caspase-mediated formation of osteoclast syncytia, in turn, was a prerequisite for the bone-resorbing activity of osteoclasts and thereby contributed to regular bone turnover.

## Results

**Fig. 1:**
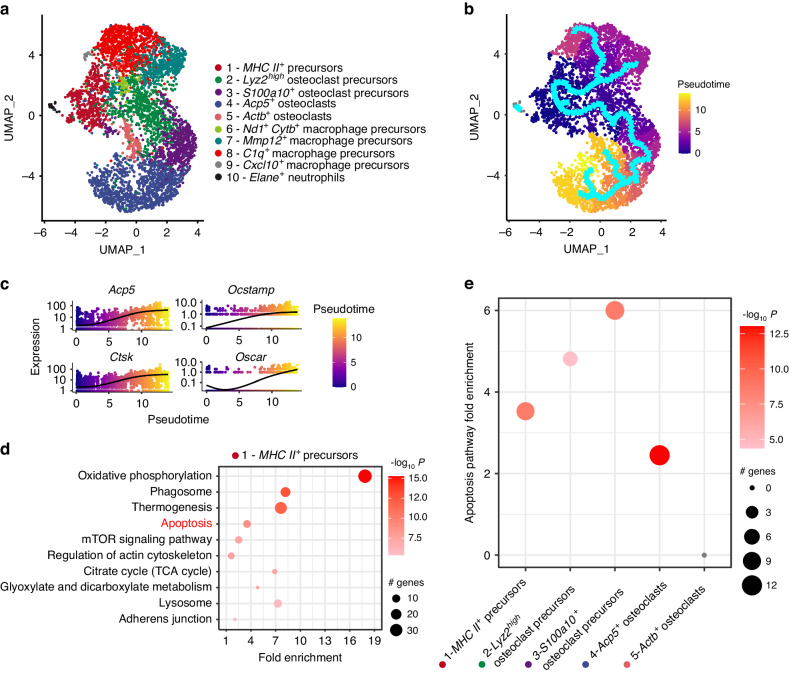
ScRNAseq profiling of differentiating osteoclast precursors. **a** Uniform manifold approximation and projection (UMAP) visualization of 10 clusters in the osteoclast culture system 3 days after initiation of RANKL-induced osteoclastogenesis. **b** Differentiation trajectory estimated by the cluster-based minimum spanning tree on a UMAP. **c** Kinetics plot representing relative expression of osteoclastic marker genes *Acp5*, *Ctsk*, *Ocstamp* and *Oscar* along the osteoclastogenesis trajectory (clusters 1, 2, 3, 4 and 5). **d** Enrichment analysis of biological processes in cells of the root monocytic osteoclast precursors cluster 1. The significant differentially regulated genes determined with Seurat were used to perform a PathfindR overrepresentation analysis using the KEGG (Kyoto Encyclopedia of Genes and Genomes) protein interaction network database. **e** Fold Enrichment of the Apoptosis pathway (KEGG mmu04210) in the clusters belonging to the osteoclastogenesis trajectory (clusters 1, 2, 3, 4 and 5)

To dissect molecular events that control differentiation and fusion of OCPs, we decided to study osteoclast differentiation via single cell RNA sequencing (scRNAseq) of bone marrow-derived OCPs. Cells were analyzed 3 days after RANKL-mediated initiation of osteoclastogenesis and thus at a stage of intermediate differentiation. This unbiased approach allowed the identification of various cellular clusters representing distinct stages of osteoclast differentiation (Fig. 1a and Fig. S1a–c). Among others, we could identify a cluster of *MHC II*^*+*^ cells that showed features of early mononuclear precursors and a cluster of *C1q*^+^ cells that displayed a gene expression signature typical of differentiated macrophages. An additional cluster of *Acp5*^*+*^ cells expressed high levels of classical osteoclast markers such as *Acp5* (encoding for TRAP), *Mmp9* (encoding for matrix metalloproteinase-9) and *Ctsk* (encoding for cathepsin K).

Pseudo-time trajectory analyses unexpectedly suggested the presence of two opposing differentiation branches where the *MHC II*^*+*^ precursors differentiated into either *C1q*^+^ macrophages or *Acp5*^*+*^ osteoclasts whereas the other clusters represented intermediate stages of the differentiation of macrophages and osteoclasts, respectively (Fig. 1b). As expected, *MHC II*^*+*^ cells gradually increased their expression of genes encoding for osteoclast markers including *Acp5*, *Ocstamp*, *Ctsk* and *Oscar* during their differentiation to *Acp5*^+^ osteoclasts (Fig. 1c).

Using this scRNAseq dataset-based pseudo-time model, we consequently sought to understand molecular mechanisms involved in the fate decision process that governed differentiation of *MHC II*^*+*^ cells into *C1q*^+^ macrophages and *Acp5*^+^ osteoclasts, respectively. A gene enrichment pathway analysis suggested apoptosis-related processes as a characteristic hallmark of differentiating osteoclasts and showed that the enrichment of apoptotic pathways gradually increased within the osteoclast differentiation branch, peaking at the stage of *S100a10*^*+*^ osteoclast precursors before decreasing again at the stage of differentiated *Acp5*^*+*^ osteoclasts (Fig. 1d, e).

**Fig. 2:**
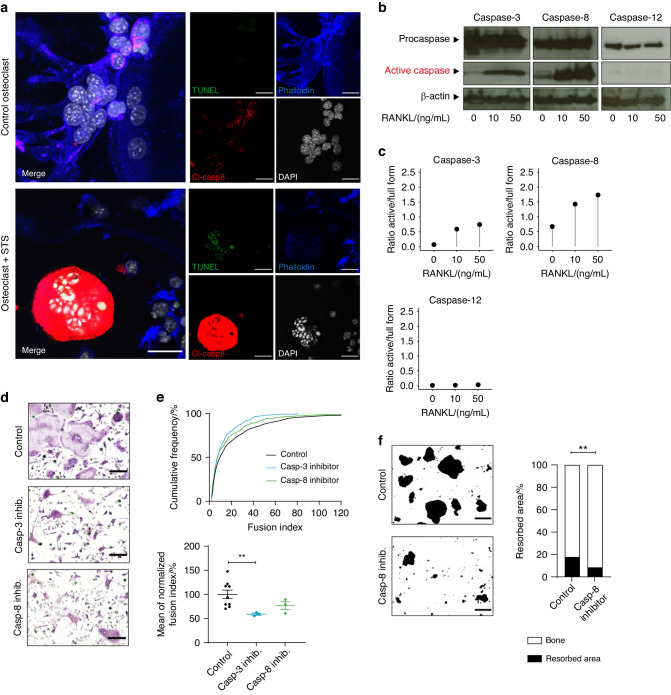
RANKL-induced caspase activity promotes fusion of osteoclast precursors. **a** Representative confocal laser scanning microscopy images of bone marrow-derived osteoclasts differentiated for 72 h in medium containing 10% L929-conditioned supernatant and 50 ng/mL RANKL either in the presence or absence of 2 μmol/L staurosporine (STS). Cells were fixed and stained for phalloidin (blue), DAPI (white), cleaved caspase-8 (“Cl-casp 8”, red) and TUNEL (green). Scale bar: 30 µm. **b** Western blot analysis of total cell extracts obtained from bone marrow-derived osteoclasts differentiated for 72 h in medium containing 10% L929-conditioned supernatant and 0, 10, or 50 ng/mL RANKL. Antibodies against the full form (“Procaspase”) or the cleaved active form (“Active caspase”) of caspases 3, 8, and 12 were used. β-actin signal served as loading control. **c** Quantification of band intensity ratios between cleaved active and full form of the Western Blot shown in **b**. **d**, **e** Bone marrow-derived osteoclasts were differentiated for 72 h in medium containing 10% L929-conditioned supernatant and 10 ng/mL RANKL in the presence of 10 μmol/L caspase-3 inhibitor, 10 μmol/L caspase-8 inhibitor, or vehicle only. Cells were fixed and stained for TRAP. **d** Representative micrographs of osteoclast size and nuclei per cell. Scale bar: 200 µm. **e** Quantification of the fusion index defined as the number of nuclei per cell. For every treatment group of the experiment, the cumulative frequency in percent of each occurring fusion index was calculated (top). The average fusion index was then determined, normalized to vehicle-only control and quantified (bottom). Indicated is mean ± SEM. Unpaired, two-tailed Student’s *t*-test, *P < 0.05; **P < 0.01; ***P < 0.001, *n* = 3 for each caspase inhibitor-treated group and *n* = 9 for control group. **f** Bone marrow-derived osteoclasts were seeded onto bone-coated osteoplates and differentiated for 72 h in medium containing 10% L929-conditioned supernatant and 10 ng/mL RANKL in the presence of 10 μmol/L caspase-8 inhibitor or vehicle only. Left panel: Representative micrographs showing resorbed area (black) *vs*. remaining bone (white). Scale bar: 200 µm. Right: Quantification of resorbed area *vs*. remaining bone area in percent. Indicated is the mean. Unpaired, two-tailed Student’s *t*-test, *P < 0.05; **P < 0.01; ***P < 0.001, *n* = 4 per group. All images and data are representative of three independent experiments

We consequently addressed a potential increase in apoptosis of RANKL-stimulated OCPs, but failed to detect classical signs of programmed cell death such as cleavage of nuclear DNA in TUNEL assays, which were otherwise evident upon stimulation with classical apoptosis-inducing agents like staurosporine (STS) (Fig. 2a). Despite the absence of RANKL-induced signs of cell death, we detected cleaved and activated caspase-8 distributed within the cytoplasm of OCPs (Fig. 2a). Western blot analyses additionally confirmed cleavage and activation of a whole subset of caspases including caspase-8 and caspase-3 in RANKL-stimulated OCPs, whereas other caspases such as caspase-12 remained intact (Fig. 2b, c). To determine whether this RANKL-mediated activation of caspases was involved in osteoclast differentiation, we blocked the activity of caspase-3 and caspase-8 by selective small pharmacological inhibitors. Although the absence of active caspases did not affect differentiation of OCPs into TRAP-positive cells, it significantly interfered with the fusion of OCPs into multinucleated syncytia (Fig. 2d, e). Caspase inhibition thereby resulted in the emergence of cells that showed a reduced content of nuclei and displayed functional alterations such as a reduced capacity to resorb bone (Fig. 2f). These findings thus suggested that RANKL-induced signaling selectively activated caspase-8 and downstream effector caspases as a part of the apoptotic machinery to induce osteoclast fusion without inducing subsequent cell death.

**Fig. 3:**
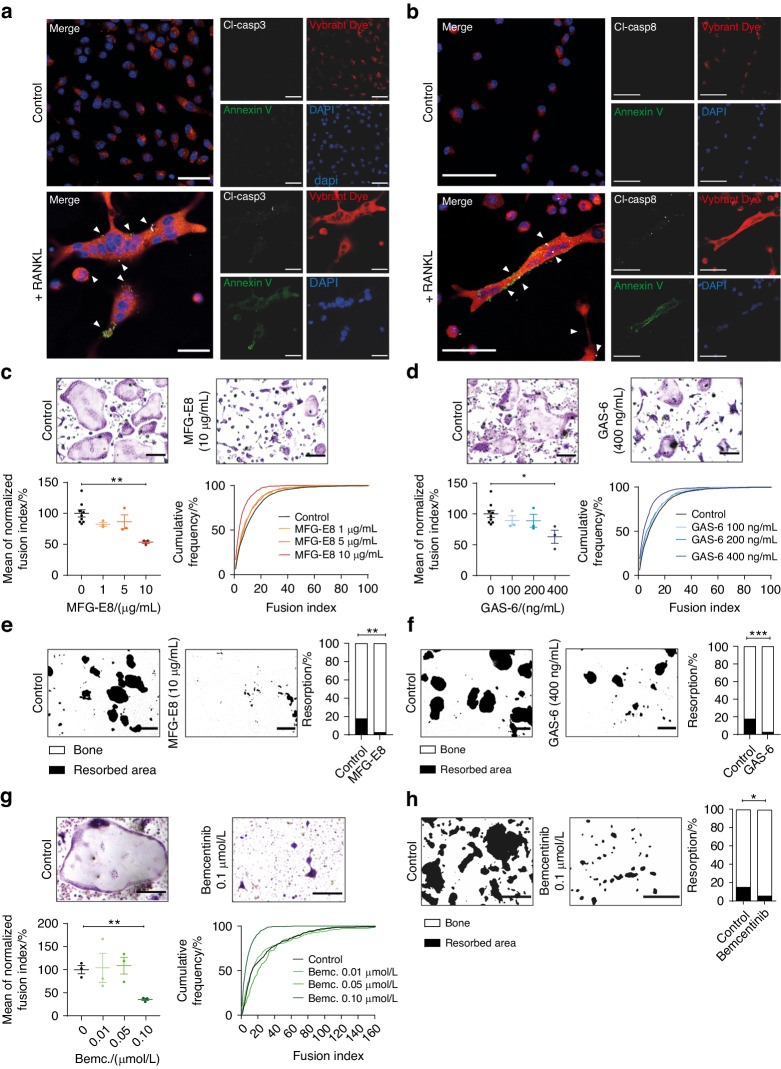
Exposure of phosphatidylserine mediates fusion of osteoclast precursors. **a**, **b** Representative confocal laser scanning microscopy images of bone marrow-derived osteoclasts differentiated for 72 h in medium containing 10% L929-conditioned supernatant either in the presence or absence of 50 ng/mL RANKL. Caspase signals are shown in white (arrowheads). Scale bar: 30 µm. **c**, **d** Bone marrow-derived osteoclasts were differentiated for 72 h in medium containing 10% L929-conditioned supernatant and 10 ng/mL RANKL in the presence of 1, 5, and 10 µg/mL MFG-E8 or vehicle only **c** or in the presence of 0, 100, 200, and 400 ng/mL GAS-6 **d**. Cells were fixed and stained for TRAP. Scale bar: 200 μm. Indicated is mean ± SEM. Unpaired, two-tailed Student’s *t*-tests, *P < 0.05; **P < 0.01; ***P < 0.001, *n* = 3 for each tested concentration of MFG-E8 or GAS-6 and *n* = 9 for control groups. **e**, **f** Bone marrow-derived osteoclasts were seeded onto bone-coated osteoplates and differentiated for 72 h in medium containing 10% L929-conditioned supernatant and 10 ng/mL RANKL in the presence of 10 µg/mL MFG-E8 or vehicle only **e** or in the presence of 0 or 400 ng/mL GAS-6 **f**. Scale bar: 200 µm. Indicated is the mean. Unpaired, two-tailed Student’s *t*-test, *P < 0.05; **P < 0.01; ***P < 0.001, *n* = 4 per group. **g** Bone marrow-derived osteoclasts were differentiated for 72 h in medium containing 10% L929-conditioned supernatant and 10 ng/mL RANKL in the presence of 0.01, 0.05, and 0.10 μmol/L Bemcentinib or vehicle only. Cells were fixed and stained for TRAP. Scale bar: 200 µm. Indicated is mean ± SEM. Unpaired, two-tailed Student’s *t*-tests, *P < 0.05; **P < 0.01; ***P < 0.001, *n* = 3 per group. **h** Bone marrow-derived osteoclasts were seeded onto bone-coated osteoplates and differentiated for 72 h in medium containing 10% L929-conditioned supernatant and 10 ng/mL RANKL in the presence of 0.1 μmol/L Bemcentinib or vehicle only. Scale bar: 200 µm. Indicated is the mean. Unpaired, two-tailed Student’s *t*-test, *P < 0.05; **P < 0.01; ***P < 0.001, *n* = 4 per group. Images and data are representative of three independent experiments except for data shown in **e**, **f**, and **h**, which are representative of two independent experiments

The process of caspase-induced apoptosis is usually paralleled by caspase-mediated disturbances of the polarity of the plasma membrane and characterized by exposure of phospholipids such as phosphatidylserine (PS) that are otherwise hidden within the plasma membrane’s inner leaflet.^8^ Such an exposure of PS is not only considered a typical hallmark of apoptosis, but additionally serves as an “eat me signal” that enables the recognition, engulfment and phagocytosis of apoptotic cells by macrophages, a process that is mediated by different types of PS receptors.^9^ We subsequently determined whether RANKL-induced caspase activation affected the composition of the plasma membrane during osteoclast differentiation. Confocal microscopy showed that the RANKL-mediated activation of OCPs indeed resulted in a rapid translocation of caspases to the cellular plasma membrane. This intracellular redistribution involved both activated initiator caspases such as caspase-8 as well as activated effector caspases such as active caspase-3 (Fig. 3a, b and Movie S1).

Co-staining with the PS-binding protein Annexin V additionally showed that these translocated membrane-associated caspases co-localized with areas of focal PS exposure (Fig. 3a, b and Movie S1). Spinning disc time-lapse confirmed PS exposure at the surface of differentiating OCPs and additionally indicated that these RANKL-induced events were usually transient and primarily localized at membrane areas that subsequently fused with neighboring precursor cells (Fig. S2a). Artificial masking of such a PS exposure with excess amounts of different soluble PS-binding molecules such as MFG-E8 or GAS-6 did not impair the differentiation of OCPs into TRAP-positive cells, but again interfered with their fusion into fully mature multinucleated osteoclasts (Fig. 3c, d). In accordance with our previous data, this inhibition of osteoclast fusion did not only result in significantly smaller TRAP-positive osteoclasts, but was additionally paralleled by a blockade of the bone-resorbing activity of the resulting osteoclast syncytia (Fig. 3e, f).

Determination of the expression levels of potential PS-recognizing surface receptors that would support such a PS-mediated fusion of OCPs showed that RANKL-stimulated OCPs rapidly upregulated the TAM receptor Axl, which is known to mediate PS recognition via GAS-6 as bridging molecule during the phagocytosis of apoptotic cells.^10^ In contrast, we observed no transcriptional regulation of its family member Tyro3 and even a downregulation of the third known TAM receptor MerTK (Fig. S2b). Bemcentinib, a small molecular inhibitor of Axl, efficiently blocked osteoclast fusion and bone resorption, whereas osteoclast differentiation was not affected (Fig. 3g, h). These findings were thus suggestive of an essential role of RANKL-induced and caspase-mediated PS exposure as well as of the PS recognition via Axl during osteoclast fusion and the generation of fully functional bone-resorbing cells.

**Fig. 4:**
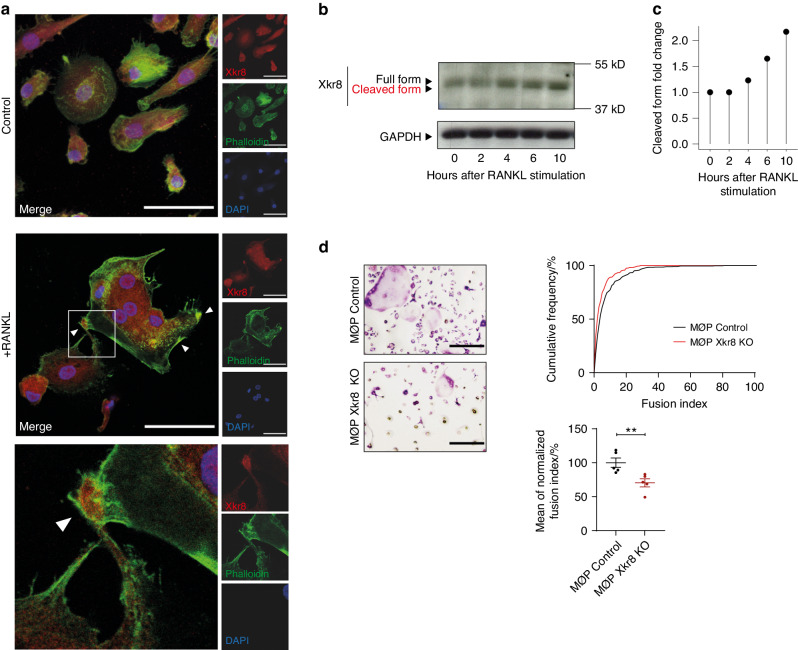
RANKL-induced osteoclast fusion is mediated via the phospholipid scramblase Xkr8. **a** Representative confocal laser scanning microscopy images of human monocyte-derived osteoclasts differentiated for 6 days in medium containing 30 ng/mL hM-CSF, 1 ng/mL hTGF-β, and 3 ng/mL hRANKL (middle and bottom) or vehicle only (top). Cells were fixed and stained for Xkr8 (red), phalloidin (green) and DAPI (blue). The white square in the middle image indicates magnified area shown on the bottom of the panel. Arrowheads indicate localization of Xkr8 at the fusion sites. Scale bar: 30 µm. **b** Western blot analysis of Xkr8 expression in cytoplasmic extracts obtained from human monocyte-derived osteoclasts differentiated for 6 days in medium containing 30 ng/mL hM-CSF, 1 ng/mL hTGF-β, and stimulated with 3 ng/mL hRANKL for 0, 2, 4, 6, and 10 h. GAPDH signal served as loading control. **c** Quantification of the fold change in cleaved band intensity relative to full form band intensity, normalized to 0 h after RANKL stimulation of the Western blot shown in **b**. **d** MØP control and Xkr8 KO MØP cells were differentiated into osteoclasts for 72 h in medium containing 10% L929-conditioned supernatant and 50 ng/mL RANKL. Cells were fixed and stained for TRAP. Left panel: Representative micrographs of osteoclast size and nuclei per cell. Scale bar: 200 µm. Right panel: Quantification of the fusion index defined as the number of nuclei per cell. For every treatment group of each experiment, the cumulative frequency in percent of each occurring fusion index was calculated (top). The average fusion index was then determined, normalized to control and quantified (bottom). Indicated is mean ± SEM. Unpaired, two-tailed Student’s *t*-test, *P < 0.05; **P < 0.01; ***P < 0.001, *n* = 5 per group. All images and data are representative of three independent experiments

During programmed cell death, caspase-induced PS exposure results from a caspase-mediated de-activation of phospholipid flippases and a caspase-mediated activation of phospholipid scramblases at the plasma membrane.^11,12^ Confocal microscopy confirmed a RANKL-mediated translocation of the phospholipid scramblase Xkr8 to the plasma membrane of OCPs where this scramblase clustered at areas of cellular fusion (Fig. 4a). Western blot analyses additionally showed the RANKL-induced cleavage of Xkr8 in osteoclast precursor cells (Fig. 4b, c), suggesting activation of its scramblase activity. To study the role of this enzyme during osteoclast fusion, we consequently performed a CRISPR/Cas9-mediated knockout of Xkr8 in a Hoxb8 conditionally immortalized OCP cell line (MØP).^13^ In accordance with a major role of Xkr8 during caspase-induced PS exposure, we observed a drastically reduced Annexin V staining on the surface of Xkr8-deficient MØP cells upon induction of apoptosis by STS (Fig. S2c, d). In accordance with our previous data, these Xkr8-deficient OCPs also displayed a significantly reduced fusion capacity in response to RANKL stimulation, whereas their differentiation into TRAP-positive cells was intact (Fig. 4d).

**Fig. 5:**
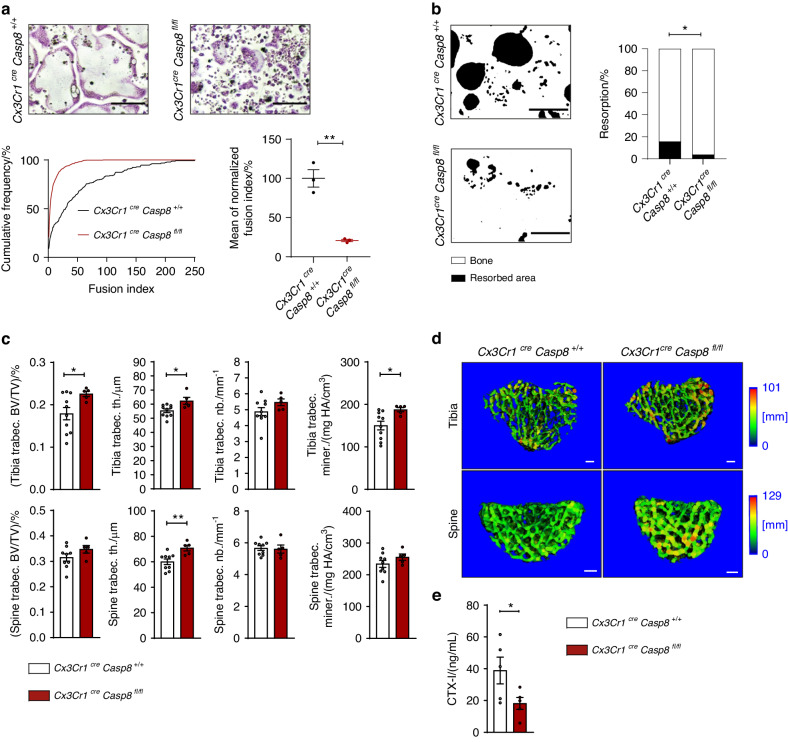
Specific deletion of caspase-8 in osteoclast precursors inhibits osteoclast fusion and bone resorption in vivo. **a** Bone marrow-derived osteoclasts obtained from *Cx3Cr1*^*cre*^*Casp8*^*+/+*^ control or *Cx3Cr1*^*cre*^*Casp8*^*fl/fl*^ littermate mice lacking caspase-8 in mononuclear phagocytes were differentiated for 72 h in medium containing 10% L929-conditioned supernatant and 50 ng/mL RANKL. Cells were fixed and stained for TRAP. Top panel: Representative micrographs of osteoclast size and nuclei per cell. Scale bar: 200 µm. Bottom panel: Quantification of the fusion index defined as the number of nuclei per cell. For both genotypes of each experiment, the cumulative frequency in percent of each occurring fusion index was calculated (left). The average fusion index was then determined, normalized to control and quantified (right). Indicated is mean ± SEM. Unpaired, two-tailed Student’s *t*-tests, *P < 0.05; **P < 0.01; ***P < 0.001, *n* = 3 per group. **b** Bone marrow-derived osteoclasts obtained from *Cx3Cr1*^*cre*^*Casp8*^*+/+*^ control or *Cx3Cr1*^*cre*^*Casp8*^*fl/fl*^ littermate mice were seeded onto bone-coated osteoplates and differentiated for 72 h in medium containing 10% L929-conditioned supernatant and 50 ng/mL RANKL. Left panel: Representative micrographs showing resorbed area (black) *vs*. remaining bone (white). Scale bar: 200 µm. Right: Quantification of resorbed area *vs*. remaining bone area in percent. Indicated is the mean. Unpaired, two-tailed Student’s *t*-test, *P < 0.05; **P < 0.01; ***P < 0.001, *n* = 4 per group. **c** µCT-based quantification of indicated bone morphometric parameters obtained from tibia (top panel) or spine (bottom panel) of 12-week-old *Cx3Cr1*^*cre*^*Casp8*^*+/+*^ control or *Cx3Cr1*^*cre*^*Casp8*^*fl/fl*^ littermate mice. BV, bone volume. TV, total volume. Trabec. th., trabecular thickness. Trabec. Nb., trabecular number. Trabec. miner., trabecular mineralization. Unpaired, two-tailed Mann-Whitney *U* test, *P < 0.05; **P < 0.01; ***P < 0.001, *n* = 10 for *Cx3Cr1*^*cre*^*Casp8*^*+/+*^ and *n* = 5 for *Cx3Cr1*^*cre*^*Casp8*^*fl/fl*^. **d** Three-dimensional µCT reconstructions showing representative cranial views of *Cx3Cr1*^*cre*^*Casp8*^*+/+*^ control or *Cx3Cr1*^*cre*^*Casp8*^*fl/fl*^ littermate-derived tibia (top panel) or spine (bottom panel). Heatmap colors indicate bone thickness in mm. Scale bar: 100 µm. **e** ELISA-based quantification of the CTX-I content of sera obtained from 12-week-old *Cx3Cr1*^*cre*^*Casp8*^*+/+*^ control or *Cx3Cr1*^*cre*^*Casp8*^*fl/fl*^ littermate mice serving as an indicator of bone resorption. Indicated is mean ± SEM. Unpaired, one-tailed Student’s *t*-test, *P < 0.05; **P < 0.01; ***P < 0.001, *n* = 5 per group. All images and data are representative of three independent experiments

To study the relevance of the identified pathway during osteoclast development in vivo, we subsequently crossed mice carrying floxed alleles of *casp8* with mice expressing a Cre-recombinase under the influence of the promoter of *Cx3cr1*. Cx3cr1 is a chemokine receptor specifically expressed in blood monocytes and embryonic precursors of tissue-resident macrophages and thus broadly active in different types of OCPs.^14^ As expected from our previous data, RANKL-stimulated OCPs isolated from *Cx3cr1*^*Cre*^*xCasp-8*^*fl/fl*^ mice differentiated into TRAP-positive cells but showed a dramatically reduced fusion capacity (Fig. 5a). Absence of caspase-8 additionally resulted in a defective bone resorbing activity of the resulting osteoclasts (Fig. 5b).

In accordance with such an impaired fusion and function of osteoclasts in caspase 8-deficient OCPs, *Cx3cr1*^*Cre*^ *Casp-8* mice also displayed an increased bone mass. μCT analysis revealed that both tibial and spinal bone showed an increase in standard parameters of bone mass such as the ratio of bone volume to total volume, trabecular thickness as well as trabecular mineralization when compared to their *Cx3cr1*^*Cre*^ *Casp-8*^*wt/wt*^ littermates (Fig. 5c, d). In addition, *Cx3cr1*^*Cre*^ *Casp-8*^*fl/fl*^ mice showed reduced levels of the collagen degradation product CTX-I in their serum, a quantitative parameter of bone resorption and osteoclast activity (Fig. 5e). These findings were thus in line with an important role of caspase-8 during the formation of fully functional bone-resorbing osteoclast syncytia and osteoclast-mediated bone resorption in vivo.

## Discussion

Our current data identify a novel RANKL-induced and caspase-8-dependent molecular pathway controlling formation of fully functional osteoclast syncytia and osteoclast-mediated bone resorption. Caspase-8 is known to act downstream of different receptors such as the TNF receptor that induce activation of NFκB. This initiator caspase acts in an autocatalytic manner resulting in its own cleavage and activation as well as cleavage and activation of downstream effector caspases including caspase-3, caspase-6 and caspase-9.^15^ Although this pathway has been originally described to induce receptor-mediated apoptosis, selective activation of caspases has been meanwhile implicated in various aspects of cell biology including inflammation, cell survival and differentiation and does not necessarily result in cell death.^16^ In epithelial cells, for instance, activation of caspase-8 is essential to prevent necroptosis as an inflammatory form of cell death and thereby supports the integrity of the intestinal barrier.^17^ Previous data have already suggested a role of caspase-3 and caspase-6 during osteoclast differentiation and bone homeostasis, although underlying molecular events and a potential role of these caspases during cellular fusion were not investigated and have thus remained elusive.^18,19^

The fact that exposure to RANKL did only activate a selected part of the apoptotic machinery in osteoclast precursors indicates a more complex scenario, where activation of caspases in osteoclast precursors is likely accompanied by the parallel activation of different regulatory pathways that prevent full blown apoptosis. This might involve members of the inhibitors of apoptosis (IAP) family of proteins, which prevent apoptosis also in the case of TNF receptor activation.^20^

Our current data show that activation of caspases in osteoclast precursors resulted in their translocation to the plasma membrane as well as catalytic cleavage and activation of the PS scramblase Xkr8. Xkr8 likewise contributes to PS exposure during apoptosis where caspase-mediated cleavage unlocks its catalytic activity and promotes PS exposure as a key signal allowing the recognition and uptake of apoptotic cells by macrophages and other phagocytic cells.^21,22^ During apoptosis, these events are paralleled by the caspase-mediated inactivation of phospholipid flippases such as ATP11C, that normally contribute to the maintenance of plasma membrane polarity by promoting PS internalization.^23^ Whether RANKL-induced signaling also results in the activation of alternative scramblases or flippases in OCPs remains to be determined. Recent data from other groups suggested a contribution of PS exposure to the fusion of osteoclasts and other multinucleated cells.^24,25^ However, exact underlying molecular events, i.e. the role of caspase-8 induced Xkr8 scramblase activation, in the PS-mediated fusion process were not investigated.

Although our data indicate a major role of the TAM receptor Axl during osteoclast fusion, several other PS binding receptors and soluble mediators might contribute to these processes in a redundant manner.^26^ Caspase-induced alterations of plasma membrane polarity and PS exposure in osteoclast precursors thus substantially change the basic biophysical properties of the plasma membrane and create ligands for cellular fusion. These fine-tuned molecular events seem to represent a conserved pathway and critical prerequisite allowing cellular fusion of osteoclasts and other multinucleated cells^27–30^ thereby affecting skeletal muscle development, fertilization and bone homeostasis.

## Material and methods

### Animals

All animal experiments were approved by the government of Middle Franconia. Mice were housed in the Franz-Penzold-Zentrum animal facility of the University of Erlangen-Nuremberg.

For in vitro osteoclast differentiation and assay, C57BL/6JRj (Stock: 000664) mice were bought from Charles River Laboratories.

*Casp8*^*fl/fl*^ mice bearing the caspase 8 floxed allele, (B6.129-*Casp8*^*tm1Hed*^/J) were purchased from Jackson Laboratory (JAX stock #027002). The exon 3 of the caspase 8 gene was flanked with loxP sites by homologous recombination.

*Cx3cr1*^*cre*^ mice (Stock: Tg(Cx3cr1^cre^)MW126Gsat/Mmucd, identification number 036395-UCD) were obtained from the Mutant Mouse Regional Resource Center (MMRC), a National Institutes of Health (NIH)-funded strain repository, and were donated to the MMRRC by the National Institute of Neurological Disorders and Stroke (NINDS)-funded GENSAT BAC transgenic project.

We crossed B6.129-Casp8^tm1Hed/J^ and Tg(Cx3cr1^cre^)MW126Gsat/Mmucd mice to obtain *Casp8*^*fl/fl*^ *Cx3Cr1*^*cre*^ mice.

### Bone marrow cell isolation, culture and differentiation

#### Mouse bone marrow monocyte-derived osteoclasts

Bone marrow was isolated from the hind long bones of control C57/BL6 mice and differentiated into osteoclasts. Shortly, femur and tibia were aseptically collected from animals at approximately 12 weeks of age. After cutting the metaphysis, the bone marrow was flushed out with sterile PBS on a cell strainer to obtain a single cell suspension. After centrifugation, erythrocyte lysis was performed using ACK Buffer and the solution was neutralized with PBS. The cell suspension was then filtrated, centrifuged again and resuspended in medium supplemented with M-CSF-conditioned media. The next day, non-adherent cells were harvested and centrifuged. Afterwards, cells were resuspended in medium containing RANKL (10 or 50 ng/mL) and M-CSF (L929 conditioned medium) and plated in 96- or 6-well plates. Control macrophages were cultured with M-CSF alone. After 2 to 5 days, cells were used for TRAP staining, RNA isolation for real-time PCR, or protein isolation for Western blot analysis.

#### Macrophage-derived osteoclasts

For *Casp8*^*fl/fl*^ *Cx3Cr1*^*cre*^ mice, bone marrow was isolated according to the previously described bone marrow isolation protocol and non-adherent cells were cultured in M-CSF containing medium for 5 days in order to increase CX3CR1 expression and thus, optimize caspase 8 deletion. After the “long M-CSF treatment”, cells were plated as described above in the presence of M-CSF and RANKL.

#### Human peripheral blood monocyte-derived osteoclasts

Peripheral Blood Mononuclear Cells (PBMC) were isolated from healthy donors and differentiated in vitro into osteoclasts. Briefly, 45 mL of blood were drawn, diluted 1:1 with PBS and layered onto 10 mL of Lymphoflot for density gradient centrifugation. The buffy coat was aspirated, transferred into a fresh tube and washed three times with ice cold 1 mmol/L EDTA-PBS. Cells were plated in the presence of hTGF-β (1 ng/mL), hM-CSF (30 ng/mL) and hRANKL (3 ng/mL) in a 96 well plate for TRAP staining or in 6-wells plates for Western blot protein sampling.

### Cell lines and culture

MØP cells: Macrophage precursor cell line (MØP). Briefly, MØP cells were generated by the Taylor Lab using a MMLV-derived retroviral vector which expresses an estrogen-dependent Hoxb transcription factor (Rosas et al.^13^) from a mouse constitutively expressing Cas9. Cells were maintained in culture in RPMI supplemented with 10% FCS and 1% Penicillin/Streptomycin in the presence of GM-CSF (10 ng/mL) and β-oestradiol (1 μmol/L). For osteoclast differentiation, MØP cells were pelleted, washed and resuspended in medium containing RANKL (50 ng/mL) and M-CSF (L929 conditioned media), and plated in 96- or 6-well plates. Control macrophages were cultured with M-CSF only. After 5 to 7 days, cells were used for either TRAP staining or apoptosis assay.

MØP CRISPR/Cas 9 knockout: MØP Cas 9 cells were transduced with a gRNA pair targeting mXkr8 via a lentiviral vector in order to generate MØP Xkr8 knockout cell line. After selection, cells were differentiated into osteoclasts according to the description above.

### Bone analyses

#### Ex vivo bone density assessment and imaging

For each mouse, one tibia and the vertebral were prepared from the mice and fixed overnight at room temperature in 4% paraformaldehyde solution in PBS. The next day, samples were transferred to a 70% ethanol solution and analysed by µCT.

Bone density was measured with a SCANCO Medical µCT 40 scanner to produce the images and analysed with SCANCO evaluation software for segmentation, 3D morphometric analysis, density and distance parameters.

### Immunofluorescence staining

For staining of coverslip-cultured osteoclasts, samples were permeabilized with 0.1% Triton X-100 in PBS for 30 min at room temperature and blocked with 0.2% BSA in PBS for 1 h. For immunofluorescence staining, the antibodies listed in the antibody table were used. Stainings were performed for 1 h at room temperature using the indicated antibodies diluted 1:100 in blocking solution. Active Caspases stainings were performed with antibodies from the Apoptosis Kit. Unbound primary antibodies were washed off with blocking solution and unlabeled primary antibodies were counterstained with donkey anti-Rabbit IgG AF594 or AF647 antibody in blocking solution for 1 h at room temperature and washed with 0.05% Tween-20 in PBS. Samples were stained with DAPI (1:2 000 dilution), washed two times with PBS, and embedded onto a glass slide with Fluorescence Mounting Medium.

### Confocal laser scanning microscopy

For high-magnification imaging of osteoclasts, a Leica TCS SP 5 II confocal microscope with acousto-optic tunable filter and acousto-optical beam splitter was used. Imaging of coverslip-cultured samples was performed using an HCX PL APO 63× glycerol objective. Fluorescence signals were generated via sequential scans, exciting Vybrant Dye Red or Alexa Fluor 594 using a diode-pumped solid-state laser (DPSS) at 561 nm, Alexa Fluor 488 or FITC-labeled staining using an argon laser at 488 nm for excitation. A third imaging sequence involved a simultaneous excitation of DAPI with a 405-nm argon laser and of Alexa Fluor 647 with a 633-nm helium-neon laser. Generated images were projected on the z-axis with ImageJ software.

### Cytochrome C quantification

The generated images were visualized and quantified with Imaris software. The amount of cytochrome C localized in cells was determined by volume rendering of the fluorescence signal for the corresponding channel and the phalloidin channel and overlapping volumes were calculated for each condition.

### Spinning disk confocal microscopy

For spinning disk confocal microscopy of histological joint sections, an inverted Zeiss Spinning Disc Axio Observer.Z1 with a Yokogawa CSU-X1M 5000 spinning disk unit, a LD C-Apochromat 63× water immersion objective (NA 1.15) and an Evolve 512 EMCCD camera was used. Fluorescence signal of pSIVA (AF488) was excited and detected at λex: 488 nm DPSS laser and λem: 525/50 nm BP filter. Acquired images were processed via Zen Blue 2.3 image acquisition software.

### Enzyme-linked immunosorbent assay

For serum analysis, blood was harvested from mice by cardiac or submandibular vein puncture and serum was separated with serum separation tubes. For cell culture supernatant analysis, culture supernatants were harvested and cleared by centrifugation before analysis. All ELISAs were performed according to the instructions of the respective manufacturer’s protocol.

### Plasma membrane extracts – protein extraction

The extraction and purification of plasma membrane and cytoplasmic protein fraction was performed using the Plasma Membrane ProteoExtract Kit, according to the manufacturer’s protocol.

### Western blotting

For murine osteoclast differentiation, cells were plated at 3 × 10^6^ cells per well in a 6-well plate. After 72 h of osteoclastogenic differentiation, cells were washed with ice-cold PBS, harvested for sampling by lysis in SDS sample buffer containing β-mercaptoethanol and denaturated at 95 °C for 10 min.

For human osteoclast differentiation, cells were plated at 6 × 10^6^ cells per well in a 6-well plate. After 7 days of differentiation, cells were washed once with ice-cold PBS and harvested for sampling in PBS by scraping the bottom of the well using a plastic policeman. Protein fractions were extracted using the ProteoExtract Kit. To adjust protein concentration, extracts were quantified with the Pierce BCA Protein-Assay Kit according to the manufacturer’s instructions and denatured in SDS sample buffer containing β-mercaptoethanol.

Protein extracts were separated on 12% SDS–polyacrylamide gels and transferred to nitrocellulose membranes. Membranes were blocked with 5% milk powder in Tris-buffered saline solution with 0.05% Tween-20 for 1 h. Blots were probed overnight with antibodies against specific full-length caspases, cleaved caspases, Xkr8, GAPDH or β-actin. Horseradish peroxidase–conjugated immunoglobulin G was used as a secondary antibody. HRP signals were revealed with Pierce ECL Western blotting substrate on radiography films. For sequential detections, blots were stripped with ReBlot Plus Strong Antibody Stripping Solution, blocked, and probed as described earlier.

### Statistical analyses

Datasets are shown as means ± SEM with sample sizes indicated in each legend. Outliers within datasets were excluded according to Grubb’s test for variation from a normal distribution. Values below the detection limit were defined as zero. All statistical analyses were performed using GraphPad Prism 8 either by two-tailed Student’s *t*-test or two-tailed Mann-Whitney *U* test, unless stated otherwise. Group differences were considered statistically significant when *P*-value ≤ 0.05.

### Single-cell RNA sequencing

Single cell RNA sequencing was performed on in vitro differentiated osteoclasts from a C57BL/6JRj wild-type mouse, at day 3 of culture with 10 ng/mL RANKL and 30 ng/mL. Single cells were captured with the 10X Genomics Chromium system. Sequencing library was generated using the 10X Genomics Single Cell 3’ Solution kit. Sequencing was performed with an Illumina sequencing system (HiSeq 4000) according to manufacturer’s protocol. Alignment and quantification of sample count matrices were performed using the 10X Genomics Cell Ranger pipeline.

### Computational analysis of single-cell RNA sequencing data

Computational analysis was performed with R GNU (version 4.0.3) using Seurat R package (3.2.3). During quality control, cells expressing more than 10% mitochondrial gene reads and less than 800 features were excluded. Additionally, cells containing between 300 and 25 000 RNA transcript counts were selected for analysis. 4 497 cells remained for analysis. Cell cycle phase scoring and regression were performed in order to mitigate the effects of cell cycle heterogeneity. Read counts per cell were normalized and scaled using the regularized negative binomial regression and variable features identified via the SCTransform function. The first 18 principal components were retained for clustering using the ElbowPlot function. Cells were clustered using a graph-based shared nearest neighbor (SNN) approach, dimensionally reduced and visualized with a Uniform Manifold Approximation and Projection (UMAP) at a resolution of 0.5.

The significantly differentially regulated marker genes for each cluster present in at least 25% of all cells were identified by Wilcoxon rank-sum test, with an adjusted *P* value < 0.05 by Bonferroni correction via the FindAllMarkers function. Gene expression was visualized for single cell on UMAP plot using the FeaturePlot function.

Enrichment analysis utilizing active subnetwork was performed using the PathfindR package (1.6.0), KEGG protein interaction network (Kyoto Encyclopedia of Genes and Genomes) and murine mmuKEGG database reference.

Construction of single-cell trajectories, identification of genes changing as a function of pseudotime and clustering of genes by pseudotemporal expression patterns were performed using the Monocle3 package (0.2.3.0). Seurat object was transferred into the Monocle3 analysis using the SeuratWrappers package (0.3.0) for the pseudotime analysis. Pseudotime calculations were performed on the top 1 000 differentially expressed genes between clusters.

## Supplementary information

Supplemental Movie S1

Supplemental Information

## Data Availability

All data and materials are available upon request.
