# Supplementary material for: Caspase-8 promotes scramblase-mediated phosphatidylserine exposure and fusion of osteoclast precursors
Source: Bone Res. 2024 Jul 11;12:40. doi: 10.1038/s41413-024-00338-4 (PMC11237014; doi:10.1038/s41413-024-00338-4)
Supplement: Supplementary file 2 — Supplemental Information [file 41413_2024_338_MOESM2_ESM.docx]

**Supplementary information to:**

*Caspase-8 promotes scramblase-mediated phosphatidylserine exposure and fusion of osteoclast precursors*

Brenda Krishnacoumar, Martin Stenzel, Hilal Garibagaoglu, Yasunori Omata, Rachel L. Sworn, Thea Hofmann, Natacha Ipseiz, Magdalena A. Czubala, Ulrike Steffen, Antonio Maccataio, Cornelia Stoll, Christina Böhm, Martin Herrmann, Stefan Uderhardt, Robert H. Jenkins, Philip R. Taylor, Anika Grüneboom, Mario M. Zaiss, Georg Schett, Gerhard Krönke and Carina Scholtysek

***Table of content:***

Supplemental Figures

*Supplemental Figure 1*

*Supplemental Figure 2*

*Description of Supplemental Movie S1*

Supplemental Material

*Table 1: Software*

*Table 2:* *Devices*

*Table 3:* *Cell culture Media, reagents and cytokines*

*Table 4:* *Kits*

*Table 5:* *Antibodies and staining reagents*

*Table 6:* *Real-time PCR primers*

*Table 7:* *Genotyping primers*

*Table 8:* *gRNA sequences and lentiviral vectors*

*Table 9: Plasmids*

**
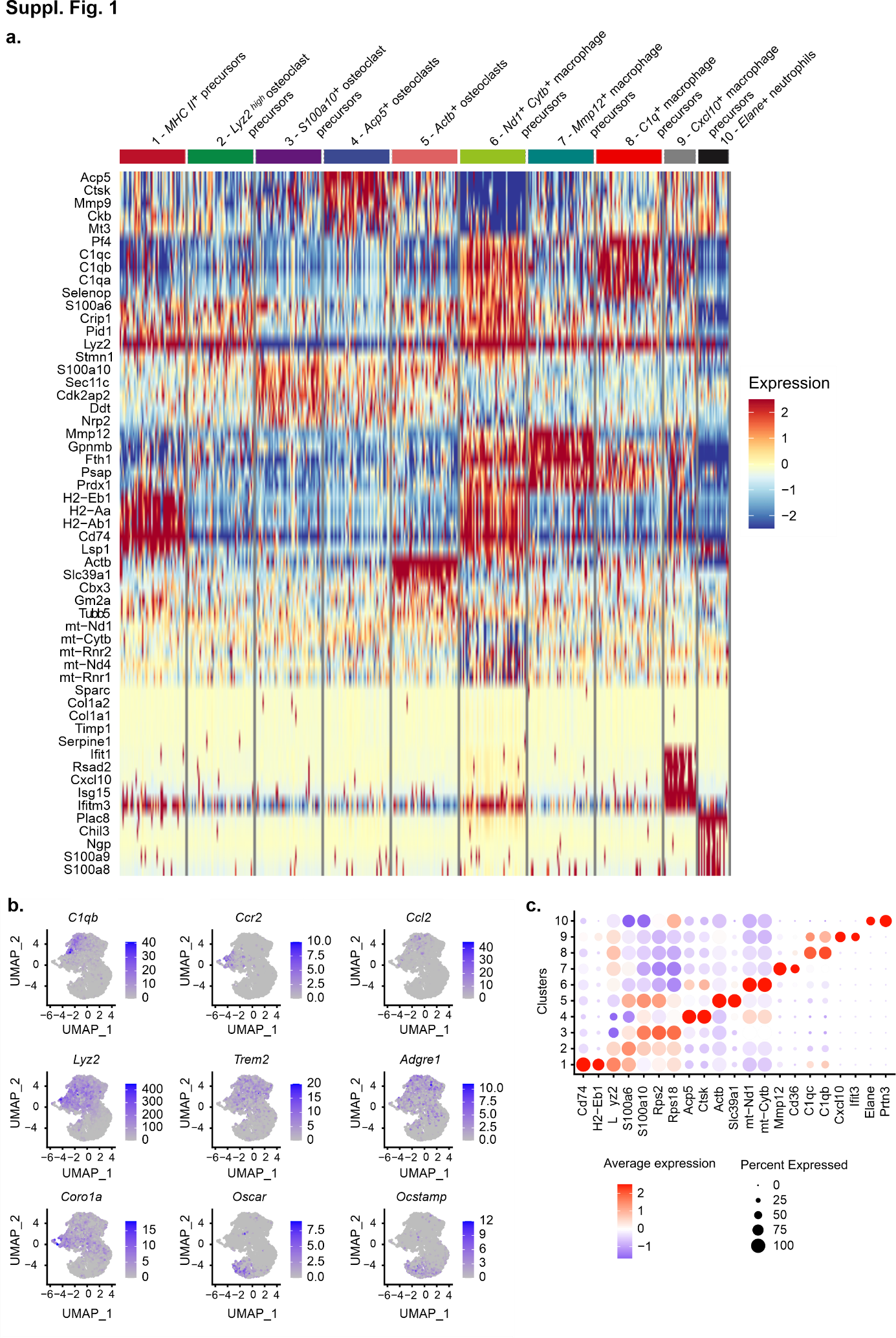
**

**Supplemental figure 1**

1. Heatmap of top 5 marker genes differentially expressed in each of the clusters indicated.
2. Expression level of the indicated marker genes overlaid onto the UMAP visualization. Dark blue, strong expression; light grey, weak expression.
3. Dot plot indicating percent of cells expressing the marker gene indicated below (dot size) alongside the average expression level for each of the clusters described in a. Red, high expression; blue, low expression.

**
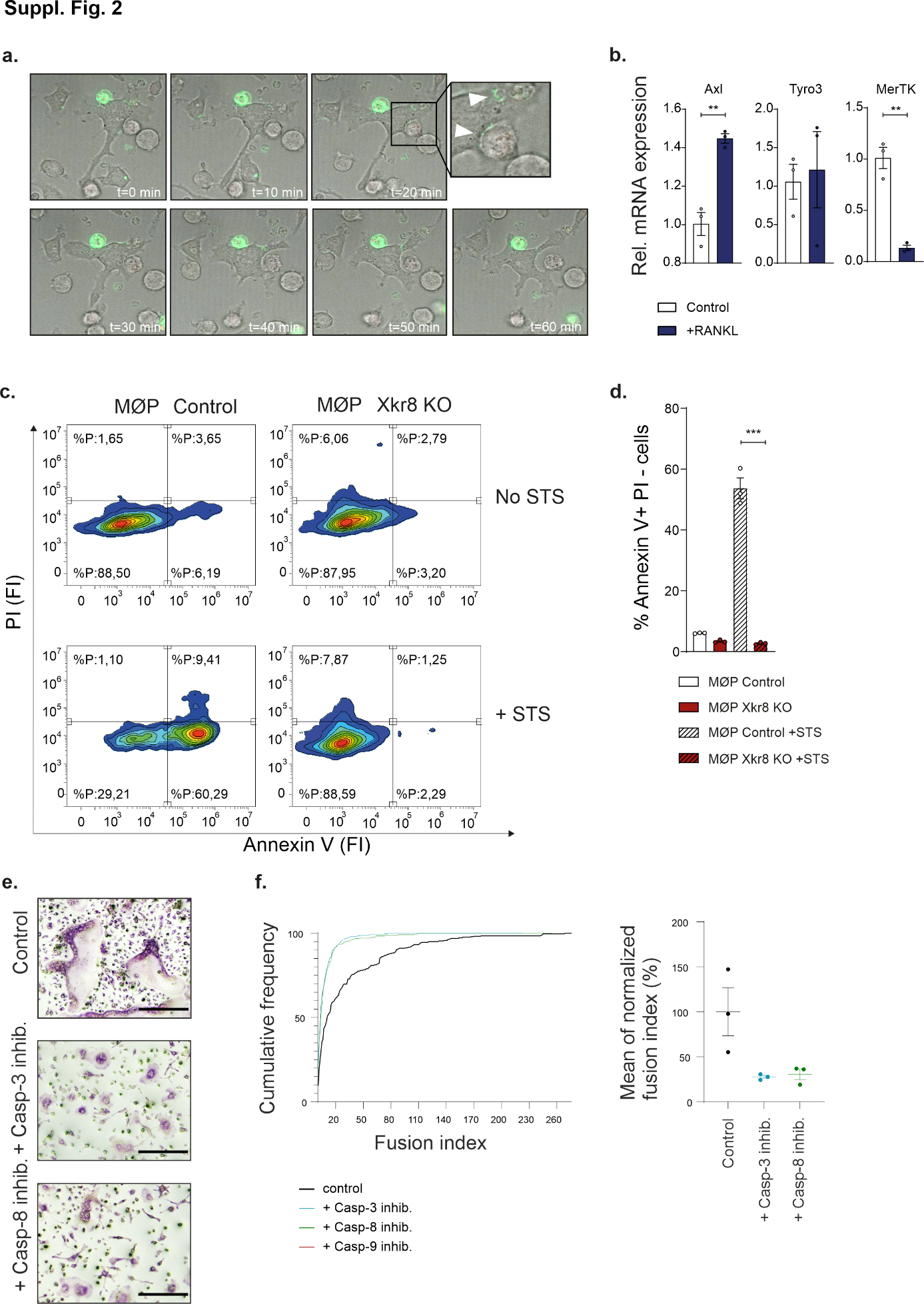
**

**Supplemental figure 2**

1. Time-lapse imaging of bone marrow-derived CD115^+^ cells differentiated into osteoclasts and stained with pSIVA (green) upon RANKL stimulation (50 ng/mL for 24 h).
2. Real-time PCR analysis of mRNA expression in osteoclasts after 72 h of RANKL stimulation. Quantification of genes *Axl, Tyro3* and *MERTK* normalized to β-actin. Indicated is mean ± SEM. Unpaired, two-tailed Student’s t-tests, *p < 0.05; **p < 0.01; ***p < 0.001, n = 3 per group.
3. Flow cytometry analysis of control and Xkr8 K MØP after induction of apoptosis for 2 h with 2 µM staurosporine (+STS) or control (no STS).
4. Bar graph showing the percentage of annexin V^+^ PI^-^ cells under indicated conditions. Indicated is mean ± SEM. Unpaired, two-tailed Student’s t-tests, *p < 0.05; **p < 0.01; ***p < 0.001, n = 3 per group.
5. MØP cells were differentiated for 72 h in medium containing 10% L929-conditioned supernatant and 50 ng/mL RANKL in the presence of caspase-3, -8 inhibitors or vehicle only (control). Cells were fixed and stained for TRAP. Representative micrographs of osteoclast size and nuclei per cell. Scale bar: 200 µm. n = 3 per group.
6. Quantification of the fusion index defined as the number of nuclei per cell from **e**. For every treatment group of each experiment, the cumulative frequency in percent of each occurring fusion index was calculated (left). The average fusion index was then determined, normalized to control and quantified (right). Indicated is mean ± SEM.

**Suppl. Movie S1:** Z-stack video of Fig. 3b.

Table 1: Software

| Software | Manufacturer | Version |
| --- | --- | --- |
| **GraphPad Prism** | GraphPad Software, Inc. (San Diego, CA, USA) | 8.3.0 |
| **Serial Cloner** | Serial Basics | 2.5 |
| **Clone Manager Professional Edition** | Sci Ed Software LLC (Westminster, CO, USA) | 9.2 |
| **CytExpert** | Beckman Coulter (Brea, CA, USA) | 2.2.0.97 |
| **FlowLogic** | Miltenyi Biotec (Auburn, CA, USA) | 7.2.1 |
| **Osteomeasure** | OsteoMetrics (Decatur, GA, USA) | 4.1.0.2 |
| **Bitplane Imaris** | Oxford Instruments, (Oxon, UK) | 10.1 |
| **Cellsens Entry** | Olympus Life Science (Tokyo, Japan) | 1.3 |
| **ImageJ** | National Institute of Health (Bethesda, MD, USA), Laboratory for Optical and Computational Instrumentation (Madison, WI, USA) | 1.52p |

Table 2: Devices

| Devices | Manufacturer |
| --- | --- |
| **SP5 Confocal Microscope SP5 II Fast Resonant Scanner** | Leica Microsystems (Wetzlar, Germany) |
| **SP8 Confocal Microscope** | Leica Microsystems (Wetzlar, Germany) |
| **Spinning Disc Microscope Axio Observer Z1** | Carl Zeiss (Jena, Germany) |
| **µCT 40** | SCANCO Medicals (Brüttisellen, Switzerland) |
| **CytoFLEX S Flow Cytometer** | Beckman Coulter (Brea, CA, USA) |

Table 3: Cell culture Media, reagents and cytokines

| Reagent | Manufacturer | Reference |
| --- | --- | --- |
| **Recombinant murine RANKL** | R&D Systems (Mineapolis, MN, USA) | 462-TR-010 |
| **Recombinant Human M-CSF** | Peprotech Inc. (Rocky Hill, NJ, USA) | 300-25-10 |
| **Recombinant Human sRANK Ligand** | Peprotech Inc. (Rocky Hill, NJ, USA) | 310-01 |
| **Recombinant Human TGF-ß3** | Peprotech Inc. (Rocky Hill, NJ, USA) | 100-36E |
| **Recombinant Mouse MFG-E8 Protein** | R&D Systems (Mineapolis, MN, USA) | 2805-MF-050 |
| **Z-DEVD-FMK Caspase-3 Inhibitor** | Selleck Chemicals (Houston, TX, USA) | S7312 |
| **Caspase-8 Inhibitor** | ENZO Life Sciences Inc. (Farmingdale, NY, USA) | ALX-260-144-R100 |
| **Caspase-8 Inhibitor Z-IETD-FMK** | Selleck Chemicals (Houston, TX, USA) | S7314 |
| **Caspase-3 and 7 Inhibitor** | ENZO Life Sciences Inc. (Farmingdale, NY, USA) | ALX-260-141-R100 |
| **Recombinant Mouse MFG-E8 Protein** | R&D Systems (Mineapolis, MN, USA) | 2805-MF-050 |
| **Recombinant Mouse GAS-6 Protein** | R&D Systems (Mineapolis, MN, USA) | 986-GS-025 |
| **Bemcentinib (R428)** | Selleck Chemicals (Houston, TX, USA) | S2841 |

Table 4: Kits

| Kit | Company | Reference |
| --- | --- | --- |
| **TRAP staining kit** | Sigma-Aldrich (Saint-Louis, MO, USA) | 387A |
| **Fast Western Blot Kits, ECL Substrate** | ThermoFischer Scientific™ (Carlsbad, CA, USA) | 35050 |
| **RatLaps CTX-I ELISA** | Immuno Diagnostic Systems (Boldon, UK) | AC-06F1 |
| **ProteoExtract Transmembrane Protein Extraction Kit** | Merck (Darmstadt, Germany) | 71772 |
| **In Situ Cell Death Detection Kit** | Sigma-Aldrich (Saint-Louis, MO, USA) | 11684795910 |
| **InFusion Kit** | Takara (Tokyo, Japan) | 121416 |
| **PEI transfection reagent** | Invitrogen™ (Waltham, MA, USA) | BMS1003-A |
| **Pierce™ ECL Western Blotting Substrate** | ThermoFischer Scientific™ (Carlsbad, CA, USA) | 32109 |

Table 5: Antibodies and staining reagents

| Antibody/reagent | Company | Reference | Dilution |
| --- | --- | --- | --- |
| **Annexin V FITC** | Biolegend (San Diego, CA, USA) | 640906 | 1:100 |
| **Phalloidin-Alexa Fluor 488/647** | Invitrogen™ (Waltham, MA, USA) | A12379/ A22287 | 1:200 |
| **Apoptosis Antibody Sampler Kit** | Cell Signaling Technology (Danvers, MA, USA) | 9930 | 1:500 for WB  1:100 for IF |
| **Rabbit anti-β-actin** | Sigma-Aldrich (Saint-Louis, MO, USA) | A2066 | 1:2000 |
| **Rabbit anti-GAPDH** | Cell Signaling Technology (Danvers, MA, USA) | 2118S | 1:10000 |
| **Rabbit anti-human XKR8 polyclonal antibody** | MyBioSource Inc. (San Diego, CA, USA) | MBS7044320-0.1 | 1:500 for WB  1:100 for IF |
| **Anti-Cytochrom C** | Life Technologies GmbH (Darmstadt, Germany) | MA3-38200-A647 | 1:200 |
| **Donkey anti-rabbit IgG-HRP** | Agilent Dako (Santa Clara, CA, USA) | P0448 | 1:2000 |
| **Goat anti-rabbit IgG Alexa Fluor 647** | Invitrogen™ (Waltham, MA, USA) | A27040 | 1:200 |
| **Goat anti-Rabbit IgG Alexa Fluor 594** | Invitrogen™ (Waltham, MA, USA) | A-11037 | 1:200 |
| **pSIVA Kit** | Abcam (Cambridge, UK) | ab129817 | 10 µL/mL |
| **Vybrant™ Multicolor Cell-Labeling Kit** | Invitrogen™ (Waltham, MA, USA) | V22889 | 5 µL/mL |
| **FcBlock** **FcR Blocking Reagent, mouse** | Miltenyi Biotec (Auburn, CA, USA) | 130-092-575 | 1:100 |
| **MACS Anti-PE MicroBeads** | Miltenyi Biotec (Auburn, CA, USA) | 130-048-801 | 20 µL for 10^7^ cells |

Table 6 : Real-time PCR primers

| Target | Forward primer sequence | Reverse primer sequence |
| --- | --- | --- |
| **β-actin** | TGTCCACCTTCCAGCAGATGT | AGCTCAGTAACAGTCCGCCTAGA |
| **MerTK** | AAGTGGGAAGAGACCGAGCTA | GCAACAGGAGGTAGGAGCTTT |
| **Axl** | CTGAAGACCCCCTCACCTTG | GCTAACGTTCTCAGGGGGAC |
| **Tyro3** | AGGCCACATTGGATAGCCTG | CCACTTTCACGAAGGAGCCA |

Table 7: Genotyping primers

| Gene | Forward sequence (5' → 3') | Reverse sequence (3' → 5') |
| --- | --- | --- |
| **Caspase 8 fl** | GAGAATATAATTCCCCCA AATCCTC | AGTCACAGCAGGGCTCACT |
| **Cre screening** | AGGTGTAGAGAAGGCACTTAGC | CTAATCGCCATCTTCCAGCAGG |

Table 8: gRNA sequences and lentiviral vectors

| gRNA | Sequence |
| --- | --- |
| **Xkr8 gRNA Strand 1** | GTGTCTGGGGGCCGCACTCG |
| **Xkr8 gRNA Strand 2** | ATGTATGCTAGTCGCACGGA |

Table 9: Plasmids

| Plasmids |
| --- |
| **pLCreW rCD2** |
